# The misuse of prescription stimulants: between overdiagnosing and malingering

**DOI:** 10.3325/cmj.2025.66.307

**Published:** 2025-10

**Authors:** Ivana Todorić Laidlaw, Sandra Caratan, Marija Štracak

**Affiliations:** 1 Department of Psychiatry, Vrapče University Psychiatric Hospital, Zagreb, Croatia; 2 Department of Psychiatry, Sv. Ivan University Psychiatric Hospital, Zagreb, Croatia; 3 Department of Psychiatry, Sisak General Hospital, Sisak, Croatia

The Diagnostic and Statistical Manual of Mental Disorders (DSM) defines attention deficit hyperactivity disorder (ADHD) as a neurodevelopmental condition marked by ongoing patterns of inattention, hyperactivity, and impulsive behavior that interfere with everyday functioning and developmental progress (1). While ADHD impacts millions globally, diagnostic rates have risen substantially over recent decades. Many elderly individuals experience high levels of ADHD symptomatology along with functional limitations and medical complications, often remaining undiagnosed and untreated (2). Both persistent adult ADHD (originating in childhood) and symptomatic adult ADHD (irrespective of childhood onset) show declining prevalence with advancing age. When adjusted to match the 2020 global demographic distribution, persistent adult ADHD prevalence stood at 2.58%, while characteristic adult ADHD reached 6.76%, which corresponds to 139.84 million and 366.33 million affected adults worldwide, respectively (3). Increased prevalence of ADHD is a result of improved diagnostic criteria, growing awareness, and changes in educational and social expectations. The rapid rise in diagnoses has also provoked suspicion and debate about the validity of some ADHD claims (4). This increase has also led to growing concerns about the potential misdiagnosis and intentional faking of ADHD symptoms (5).

The updated European Consensus Statement on diagnosis and treatment of adult ADHD advocates for screening adults who present with a background of inattentiveness, hyperactivity/impulsivity, emotional dysregulation, additional psychiatric conditions, conduct issues, criminal involvement, or familial ADHD diagnoses. The diagnostic process should be conducted in specialized clinical settings and rely on multiple information sources, including patient self-reporting, family member accounts, and available documentation such as school records. Several validated diagnostic interviews exist for adult populations. The semi-structured Diagnostic Interview for ADHD in adults has been validated against both DSM-IV-TR and DSM-5 criteria, while the Conner's Adult ADHD Diagnostic Interview for DSMV follows DSM-IV guidelines. Among validated screening instruments used in adults, the Adult ADHD Self-Report Scale serves as a standard screening measure. Parental reports of ADHD symptoms tend to be understated relative to self-reported data. Recall bias has historically led to the exclusion of older participants from ADHD investigations. Self-report instruments lack sufficient sensitivity to identify fabricated ADHD presentations, as individuals without genuine symptoms can easily manipulate these assessments, which may yield elevated false-positive rates (6). Alternative strategies to the age of onset requirement include verifying symptom continuity throughout multiple years, documenting their presence across developmental stages, and incorporating supplementary data along with educational records (2).

The primary pharmacological agents in prescription stimulant treatment for ADHD are methylphenidates and dextroamphetamine-amphetamine. These medications are designated as controlled substances and are frequently prescribed. While therapeutically effective for ADHD symptom management when administered appropriately, they carry inherent misuse liability (6).

The non-medical utilization of stimulants represents an ongoing public health challenge. Motivations for non-prescribed stimulant use divide roughly equally between recreational purposes (achieving euphoria or intoxication) and performance optimization (increasing productivity and maintaining wakefulness) (7).

Acquiring stimulants through feigning ADHD symptoms occurs more frequently than formerly recognized. According to a nationally representative adult survey, approximately 20% of past-year nonmedical users exaggerated symptoms to secure prescriptions. Documented motivations for ADHD malingering include acquiring stimulant prescriptions for cognitive enhancement, accessing supplemental academic services and legal accommodations (isolated testing spaces, extended examination periods, homework reductions, and note-taking assistance), recreational consumption, or illicit market distribution (7).

Such misuse creates genuine safety hazards, including overdose risk and adverse drug-drug interactions with prescribed or illicit substances. Illicit users typically lack knowledge of a medication's official contraindications, warnings, or interaction profiles. Numerous adverse reactions have been documented with nonmedical prescription stimulant use, particularly when combined with recreational substances like alcohol. As ADHD prescription volumes increase, diversion for nonmedical purposes – costing health care payers millions annually – will likely escalate proportionally (7).

During the past ten years, both ADHD diagnoses and prescription stimulant utilization have increased sharply among pediatric and adult populations. The most reported motivation for misuse was help with alertness and concentration. Frequently, abusers use prescription stimulants from friends who got a prescription from a health care provider (4). Prescription stimulants have addictive potential, and malingering is almost undetectable. Clinicians should make an effort to reconsider the pattern of prescribing stimulants. Malingering should be added to the differential diagnosis if suspected. Diagnostic assessment of ADHD should include more collateral information from different sources, medical history, forensic history, and clarifying secondary gains. When the diagnosis is unclear, clinicians should wait before prescribing a stimulant and spend more time completing a full ADHD assessment (6). Psychoeducational and psychotherapeutic interventions could play a major role in the prevention of misuse. Increasing both clinical and public awareness through workshops and educational interventions, which could include parents, students, and educational staff, could decrease the nonmedical use of prescription stimulants. Future research based on neuroimaging methods is required to objectify ADHD symptoms and help clinicians in accurately diagnosing and avoiding the feigning of symptoms. Further studies are required for a better understanding of ADHD and malingering.
